# Sustained antiviral response against *in vitro* HIV-1 infection in peripheral blood mononuclear cells from people with chronic myeloid leukemia treated with ponatinib

**DOI:** 10.3389/fphar.2024.1426974

**Published:** 2024-09-23

**Authors:** Mario Manzanares, Fernando Ramos-Martín, Sara Rodríguez-Mora, Guiomar Casado-Fernández, Clara Sánchez-Menéndez, Alicia Simón-Rueda, Elena Mateos, Miguel Cervero, Adam M. Spivak, Vicente Planelles, Montserrat Torres, Valentín García-Gutiérrez, Mayte Coiras

**Affiliations:** ^1^ Immunopathology and Viral Reservoir Unit, National Center of Microbiology, Instituto de Salud Carlos III, Madrid, Spain; ^2^ PhD Program in Biomedical Sciences and Public Health, Universidad Nacional de Educación a Distancia (UNED), Madrid, Spain; ^3^ Biomedical Research Center Network in Infectious Diseases (CIBERINFEC), Instituto de Salud Carlos III, Madrid, Spain; ^4^ Faculty of Sciences, Universidad de Alcalá, Madrid, Spain; ^5^ Hematology and Hemotherapy Service, Instituto Ramón y Cajal de Investigación Sanitaria (IRYCIS), Hospital Universitario Ramón y Cajal, Madrid, Spain; ^6^ Internal Medicine Service, Hospital Universitario Severo Ochoa, Madrid, Spain; ^7^ School of Medicine, Universidad Alfonso X El Sabio, Madrid, Spain; ^8^ Division of Microbiology and Immunology, Department of Pathology, University of Utah School of Medicine, Salt LakeCity, UT, United States

**Keywords:** HIV-1, ponatinib, cytotoxic immunity, antiviral response, chronic myeloid leukemia

## Abstract

HIV-1 infection cannot be cured due to long-lived viral reservoirs formed by latently infected CD4^+^ T cells. “Shock and Kill” strategy has been considered to eliminate the viral reservoir and achieve a functional cure but the stimulation of cytotoxic immunity is necessary. Ponatinib is a tyrosine kinase inhibitor (TKI) clinically used against chronic myeloid leukemia (CML) that has demonstrated to be effective against HIV-1 infection *in vitro*. Several TKIs may induce a potent cytotoxic response against cancer cells that makes possible to discontinue treatment in people with CML who present long-term deep molecular response. In this longitudinal study, we analyzed the capacity of ponatinib to induce an antiviral response against HIV-1 infection in peripheral blood mononuclear cells (PBMCs) obtained from people with CML previously treated with imatinib for a median of 10 years who changed to ponatinib for 12 months to boost the anticancer response before discontinuing any TKI as part of the clinical trial NCT04043676. Participants were followed-up for an additional 12 months in the absence of treatment. PBMCs were obtained at different time points and then infected *in vitro* with HIV-1. The rate of infection was determined by quantifying the intracellular levels of p24-gag in CD4^+^ T cells. The levels of p24-gag+ CD4^+^ T−cells were lower when these cells were obtained during and after treatment with ponatinib in comparison with those obtained during treatment with imatinib. Cytotoxicity of PBMCs against HIV-infected target cells was significantly higher during treatment with ponatinib than during treatment with imatinib, and it was maintained at least 12 months after discontinuation. There was a significant negative correlation between the lower levels of p24-gag+ CD4^+^ T−cells and the higher cytotoxicity induced by PBMCs when cells were obtained during and after treatment with ponatinib. This cytotoxic immunity was mostly based on higher levels of Natural Killer and Tγδ cells seemingly boosted by ponatinib. In conclusion, transient treatment with immunomodulators like ponatinib along with ART could be explored to boost the antiviral activity of cytotoxic cells and contribute to the elimination of HIV-1 reservoir.

## 1 Introduction

The use of antiretroviral therapy (ART) has transformed the infection by the human immunodeficiency virus type 1 (HIV-1) into a chronic disease. However, ART must be taken for life because viral rebound occurs rapidly after discontinuation (Olsen et al., 2007). The main barrier preventing the eradication of HIV-1 is the formation of long-lived reservoirs in latently infected cells, mostly CD4^+^ T−cells and macrophages (Finzi et al., 1997; Chun et al., 1997; Sonza et al., 2001). These cells contain proviruses integrated in the cellular genome that remain refractory to both ART and cellular immune response (Chun et al., 1997; Hermankova et al., 2003; Finzi et al., 1999). Latently infected cells may contain replication-competent proviruses that produce infectious viral particles upon stimulation (Blankson et al., 2002), while cells harboring defective proviruses generate viral proteins that trigger T-cell activation and a chronic systemic inflammation that is characteristic of people with HIV (PWH) (Hatano et al., 2013; Stunnenberg et al., 2020; Deeks et al., 2013). Several pharmacological strategies have been assayed to reduce or eliminate the HIV-1 reservoir, such as “Shock and Kill” that is based on the reactivation of latently infected cells by using latency reversal agents (LRAs) and the increased activity of the cytotoxic immunity against infected cells (Martrus and Altfeld, 2016; Kim et al., 2018). However, until now these strategies have not been sufficiently effective *in vivo* to induce a significant effect on the viral reservoir due to low capacity of LRAs to reactivate the reservoir and to impaired cytotoxic activity in PWH (Thomas et al., 2020; Kuang and Brockman, 2018; Ait-Ammar et al., 2019; Duggan et al., 2023). Therefore, alternative well-tolerated, effective, and affordable strategies are needed. In this regard, our group proposed the use of tyrosine kinase inhibitors (TKI) such as dasatinib and ponatinib to control HIV-1 infection and proviral replication and we described that a major mechanism of action of these drugs against HIV-1 was the preservation of the antiviral activity of SAMHD1 (Bermejo et al., 2018). SAMHD1 is a deoxynucleotide triphosphohydrolase that depletes the pool of intracellular dNTPs necessary for HIV-1 replication in noncycling cells (Wu and KewalRamani, 2006; Berger et al., 2011). SAMHD1 phosphorylation at T592 (pSAMHD1) by cyclin-dependent kinases deactivates its activity, rendering the cells permissive to HIV-1 infection (Cribier et al., 2013; Williams et al., 2022).

TKIs are immunomodulatory agents currently used in clinic for the treatment of chronic myeloid leukemia (CML) (Andreieva et al., 2016). Therefore, repositioning of drugs previously used as cancer immunotherapy may be an option to influence the viral reservoir in PWH. Moreover, certain approaches may boost the cell-mediated immunity to help eliminate HIV-1-infected cells (Bermejo et al., 2018; Kreutzman et al., 2010; Climent and Plana, 2019). The cytotoxic immunity mediated by CD8^+^ T cells, Natural Killer (NK), and Tγδ cells may be directed to both cancer cells and virus-infected cells (Mishra et al., 2014; Liu and Zhang, 2020; Raskov et al., 2020). In this regard, TKIs such as imatinib, dasatinib, and ponatinib have also sparked interest against HIV-1 infection due to they may induce the expansion of large granular lymphocytes (LGLs) with NK phenotype and sustained cytotoxic activity *in vivo* (Climent and Plana, 2019; Hayashi et al., 2012; Mustjoki et al., 2013; Schiffer et al., 2016; Rodríguez-Agustín et al., 2023) that may even persist after treatment discontinuation (Chen et al., 2021). The development of high levels of these long-lived functional cytotoxic cell populations in individuals with CML who achieved a sustained deep molecular response (DMR) has been associated with better anti-leukemic responses (Kreutzman et al., 2010; Hayashi et al., 2012; Mustjoki et al., 2013; Vigón et al., 2020) and long-term treatment-free remission (TFR) after TKI discontinuation (Cortes et al., 2019; Cortes et al., 2016).

We previously demonstrated that cytotoxic cell populations developed after treatment with dasatinib in individuals with CML had a potent antiviral activity against autologous HIV-1-infected CD4^+^ T cells that persisted at least 12 months after treatment discontinuation (Vigón et al., 2020). In this study, we recruited individuals with CML on treatment with imatinib who were treated with ponatinib for 12 months to boost the anticancer response before discontinuing treatment with any TKI as part of clinical trial NCT04043676. These individuals were then followed-up for an additional 12 months without intervention. The capacity of ponatinib, which is more potent than dasatinib (Kayabasi et al., 2022), to induce a sustained resistance against HIV-1 infection after 1-year treatment was evaluated, as well as the possible association with the development of anticancer cytotoxic immunity with antiviral activity. The results obtained could help determine the validity of using short-term treatment with TKIs in PWH as adjuvants of ART to contribute to the improvement of “Shock and Kill” strategies, as well as to reposition ponatinib as an antiviral drug against HIV-1 infection.

## 2 Materials and methods

### 2.1 Study subjects

This is a substudy of the multicenter, open-label, single-arm, Phase II exploratory trial NCT04043676 (EudraCT 2017-004565-27) that recruited 11 individuals with CML Philadelphia Chromosome-positive (Ph+) to determine the capacity of ponatinib to increase the probability of not relapse of CML after discontinuing TKI treatment, which occurs in approximately 50% of cases, as was previously described (Rousselot et al., 2014). Sample size was calculated using the sample size calculator Granmo (Marrugat et al., 1998), assuming an alpha risk of 0.05 and a statistical power greater than 80%. All participants were HIV-1 negative. The primary endpoint of NCT04043676 was to evaluate the proportion of participants without confirmed loss of MR4 (Molecular Response 4.0 log reduction from baseline) or loss of MMR (major molecular response) within the first 52 weeks following cessation of ponatinib, while the main endpoint of the Exploratory Objective of NCT04043676 that is described in the present report was to evaluate the capacity of ponatinib to induce the development of cytotoxic cell populations with anticancer and antiviral activity. The main inclusion criteria were to be over 18 years old, not being pregnant, having been on treatment with 400 mg of imatinib for at least 4 years, to present DMR4 (>4 log reduction of *BCR::ABL1*, the causative agent of CML) for at least 4 months, and to sign the informed written consent to participate in the study. Upon recruitment, the participants stopped imatinib and started an intensification treatment with 15 mg of ponatinib daily for 12 months before discontinuing treatment with any TKI. The follow-up was performed during an additional 12 months because CML relapse is expected to occur within the first 6–8 months after treatment discontinuation (Rousselot et al., 2014; Etienne et al., 2017; Saussele et al., 2018; Okada et al., 2018; Mahon et al., 2010). The study design and recruitment of participants took place in the Hospital Universitario Ramón y Cajal (Madrid, Spain), Hospital Regional de Málaga (Málaga, Spain), Hospital Universitario Virgen de la Salud (Toledo, Spain), and Hospital Universitario La Princesa (Madrid, Spain).

### 2.2 Ethical statement

All participants gave informed written consent to participate in this study that was performed in accordance with the Helsinki Declaration. The protocol was previously reviewed and approved by the Ethical Committee for Clinical Research with Medicines of Hospital Universitario La Princesa (Madrid, Spain) (protocol number 17/18) and the other participating hospitals. Anonymity and confidentiality were ensured by the Spanish and European Data Protection Acts.

### 2.3 Blood samples

Five blood samples were collected from each participant in EDTA Vacutainer tubes (Becton Dickinson, Franklin Lakes, NJ): first sample was collected before starting treatment with ponatinib (Start ponatinib: switching from imatinib to ponatinib); second sample was collected after 12 months of treatment with ponatinib (Stop ponatinib: switching from ponatinib to discontinuation); and the next three samples were collected 3, 6, and 12 months after discontinuation of ponatinib. Blood samples were immediately processed after collection and peripheral blood mononuclear cells (PBMCs) were isolated by centrifugation through Ficoll-Hypaque gradient (Sigma Aldrich, St. Louis, MO) and cryopreserved in liquid nitrogen until analysis. Due to lack of sample, not all determinations were performed in all samples.

### 2.4 HIV-1 infection of PBMCs

PBMCs were cultured in RPMI 1640 supplemented with 10% (v/v) fetal calf serum (FCS), 2 mM L-glutamine, 100 ug/mL streptomycin, 100 UI/mL penicillin (Biowhittaker, Walkersville, MD), and activated with 10 μg/mL phytohemagglutinin (PHA) (Sigma-Aldrich) and 300 units/mL IL-2 (Chiron, Emeryville, CA) for 48 h. Cells were then infected *in vitro* with HIV-1 strain NL4-3_wt (Adachi et al., 1986) by spinoculation and cultured for 72 h, as described previously (López-Huertas et al., 2011). CXCR4-tropic NL4-3_wt strain was used instead of more clinically relevant CCR5-tropic viral strains due to ponatinib impedes CCR5 expression of the cell surface upon T-cell activation, while it has no effect on CXCR4 expression levels (Bermejo et al., 2018).

### 2.5 Intracellular staining of p24-gag and pSAMHD1 in CD4^+^ T cell memory subsets

HIV-1 infection of PBMCs was evaluated by intracellular staining of the levels of p24-gag in CD4^+^ T cells. Cells were first stained on the surface to label total CD4^+^ T cells and CD4 memory subpopulations using the following antibodies: CD3-BV510, CD8-APC-H7, CD45RA-PeCy7, and CCR7-Bv421. CD3^+^CD8^−^were assumed to be CD4^+^ T cells, which included those cells with downregulated CD4 expression caused by HIV-1 infection, as described previously (Morón-López et al., 2017). CD4 memory subpopulations were determined as follows: Naïve (TN) (CD45RA + CCR7^+^); Central Memory (TCM) (CD45RA-CCR7^+^); Effector Memory (TEM) (CD45RA-CCR7^−^); and Terminally Differentiated Effector Memory (TEMRA) (CD45RA + CCR7^−^). PBMCs were then fixed and permeabilized with IntraPrep Permeabilization Reagent (Beckman Coulter Spain) and HIV-1 core antigen p24-gag was stained using a specific antibody (clone kc57) conjugated with fluorescein isothiocyanate (FITC) (Beckman Coulter Spain, Barcelona, Spain). SAMHD1 phosphorylation at T592 (pSAMHD1) was determined by using an antibody conjugated with phycoerythrin (PE) (Cell Signaling Technology Europe, Leiden, Netherlands). Isotype controls were used to determine the background signal. Data acquisition was performed using BD LSR Fortessa X-20 flow cytometer (BD Biosciences, San José, CA) with BD FACSDiva software. Gating strategy is shown in Supplementary Figure S1. Data analysis was performed with FlowJo v10.7.1 (TreeStar, Ashland, OR).

### 2.6 Characterization of cytotoxic immunity

TZM-bl/JC53BL-13 cell line (human cervix; NIH AIDS Research and Reference Reagent Program, No. 8129) was obtained from the existing collection of Instituto de Salud Carlos III (Madrid, Spain). As described previously in Casado-Fernández et al. (2024), a monolayer of TZM-bl cells was infected with HIV-1 NL4.3_wt strain for 48 h in DMEM supplemented with 10% (v/v) FCS, 2 mM L-glutamine, 100ug/mL streptomycin, 100 UI/mL penicillin (Biowhittaker, Walkersville, MD). PBMCs were added to the monolayer (1:1) and co-cultured for 1 h. TZM-bl cells were then detached with trypsin-EDTA solution (Sigma Aldrich-Merck, Darmstadt, Germany) and the induction of apoptosis was determined by quantifying caspase-3 activity in these cells with Caspase-Glo 3/7 Analysis system (Promega), based on previous reports (Jerome et al., 2003). Direct cellular cytotoxicity (DCC) fold was calculated using the following formula:

$$\frac{\begin{array}{c} Average\;caspase-3\;activity\;\left(RLUs\right)\;in\;HIV-infected\;TZM\;cocultured\;with\;PBMCs\\ obtained\;after\;ponatinib\;treatment\;and\;during\;TFR \end{array}}{\begin{array}{c} Average\;caspase-3\;activity\;\left(RLUs\right)\;in\;HIV-infected\;TZM\;cells\;cocultured\;with\;PBMCs\;\\ obtained\;before\;ponatinib\;treatment \end{array}}$$

PBMCs were recovered from supernatants after co-culture with HIV-1-infected TZM-bl cells and then stained with CD3-Bv510, CD8-APCH7, CD56-Bv605, TCRγδ-PE, and CD107a-PE-Cy7 (BD Biosciences) to determine the presence of cytotoxic cell populations such as NK, CD8, and Tγδ cells with degranulation capacity (CD107a+). Data acquisition was performed using spectral flow cytometer Cytek Aurora and SpectroFlo software (Cytek). The gating strategy is shown in Supplementary Figure S2.

### 2.7 Antiviral activity of cytotoxic cells against autologous HIV-1-infected CD4^+^ T cells

As previously described by Vigón et al. (2020), CD4^+^ T cells were isolated from PBMCs obtained 12 months after ponatinib discontinuation using CD4^+^ T−cell Isolation Kit (Miltenyi Biotec, Bergisch Gladbach, Germany). Isolated CD4^+^ T cells were activated with PHA and IL-2 for 48 h and then infected *in vitro* with NL4-3_wt for 72 h in the presence or absence of autologous cytotoxic cells (NK, CD8^+^ T cells, and Tγδ cells) (ratio 1:1). Cytotoxic cells were not further purified due to lack of sample to avoid cell loss. Intracytoplasmic synthesis of p24-gag was analyzed in CD4^+^ T cells by flow cytometry as described above.

### 2.8 Statistical analysis

Statistical analysis was performed with GraphPad Prism v10.2.1 (GraphPad Software Inc., San Diego, CA) and Stata 17 (StataCorp, College Station, TX). Analyses were performed between selected paired time points, considering samples collected while on treatment with imatinib or ponatinib as basal samples. Data normality distribution was determined with Shapiro-Wilk normality test. Paired t-test was used to calculate statistical significance when data followed a normal distribution, and Wilcoxon matched pairs signed rank test was used when data normality was not assumed. Pearson’s or nonparametric Spearman’s rank correlation coefficients (r) were calculated according to data normality to evaluate the association between intracellular levels of p24-gag and pSAMHD1. P values (*p*) < 0.05 were considered statistically significant in all comparisons.

## 3 Results

### 3.1 Participants’ characteristics

Twenty-three individuals were recruited for NCT04043676, of which 3 (13%) presented serious adverse events (AEs) related to ponatinib and discontinued treatment prematurely (Pérez-Lamas et al., 2023). Nineteen individuals (82%) entered the TFR phase, of which 14 continued with MMR without requiring treatment after a median follow-up of 12 months. Of these, blood samples from 11 individuals were available to develop the Exploratory Objective of NCT04043676 to evaluate the capacity of ponatinib to induce the development of cytotoxic cell populations with anticancer and antiviral activity which results are described in the present report.

The main sociodemographic and clinical characteristics of all participants in the substudy (n = 11) are detailed in Table 1. Most participants were male (82%) with a median age at sampling of 50 years old (Interquartile range (IQR) 40–57) and median age at CML diagnosis of 39 years old (IQR 22–43). Median time from CML diagnosis was 11 years (IQR 7–19), median time on imatinib was 10.2 years (IQR 4.7–16.3), and most participants (64%) presented low Sokal risk before starting treatment with ponatinib.

**TABLE 1:** Sociodemographic and clinical data of the participants recruited for this study.

Participant’s ID	Gender (M/F)	Age (Years)	Age at CML diagnosis (Years)	Sokal risk score before ponatinib treatment	Time from CML diagnosis (Years)	Time on treatment with imatinib (Years)	IgG against CMV
02_02	M	62	40	UD	21	18.0	Pos
02_03	M	57	51	UD	7	4.2	Pos
04_01	M	41	22	Low	20	16.3	Pos
04_02	F	53	43	Intermediate	9	4.7	Pos
04_03	M	48	30	Low	19	15.2	Pos
04_04	M	39	21	Low	18	14.5	Pos
04_06	M	29	19	Low	10	6.5	Neg
04_07	F	40	32	Low	7	6.5	Pos
04_08	M	56	51	Low	5	4.7	Pos
04_09	M	50	39	Low	11	10.2	Pos
10_01	M	59	43	UD	16	16.4	Pos

CML, chronic myeloid leukemia; F, female; M, male; Neg, negative; Pos, positive; UD, undetermined.

Ponatinib was well tolerated by all participants who finished the substudy, although they reported AEs similar to those presented by other participants in NCT04043676: constipation (30%), asthenia (26%), myalgias and arthralgias (26%), and skin rash (17%) (Pérez-Lamas et al., 2023). Most participants were seropositive for cytomegalovirus (CMV) (91%) but they did not present viral reactivation or opportunistic infections during or after treatment with ponatinib, ruling out immunosuppression during treatment. After 12 months of treatment with ponatinib, all participants met criteria to discontinue, and they stopped treatment with any TKI. They were followed-up for an additional 12 months after discontinuation to monitor relapse of CML. None of the participants in the substudy relapsed of CML and they still maintained DMR after a median time of 2.9 years (IQR 2.24–3.51) since ponatinib discontinuation.

### 3.2 Resistance to HIV-1 infection *in vitro* after treatment with ponatinib

Treatment with ponatinib did not significantly affect the levels of CD4^+^ T cells in blood. Median percentage of CD4^+^ T cells was 33.3% (IQR 18.7–44.1) during treatment with imatinib. After 1 year of treatment with ponatinib, median percentage of CD4 was 32.0% (IQR 15.6–45.1), and 3, 6, and 12 months after treatment discontinuation, median percentage of CD4^+^ T cells was 33.0% (IQR 20.8–41.8), 34.6% (19.6–46.7), and 30.8% (16.9–42.4).

**FIGURE 1:**
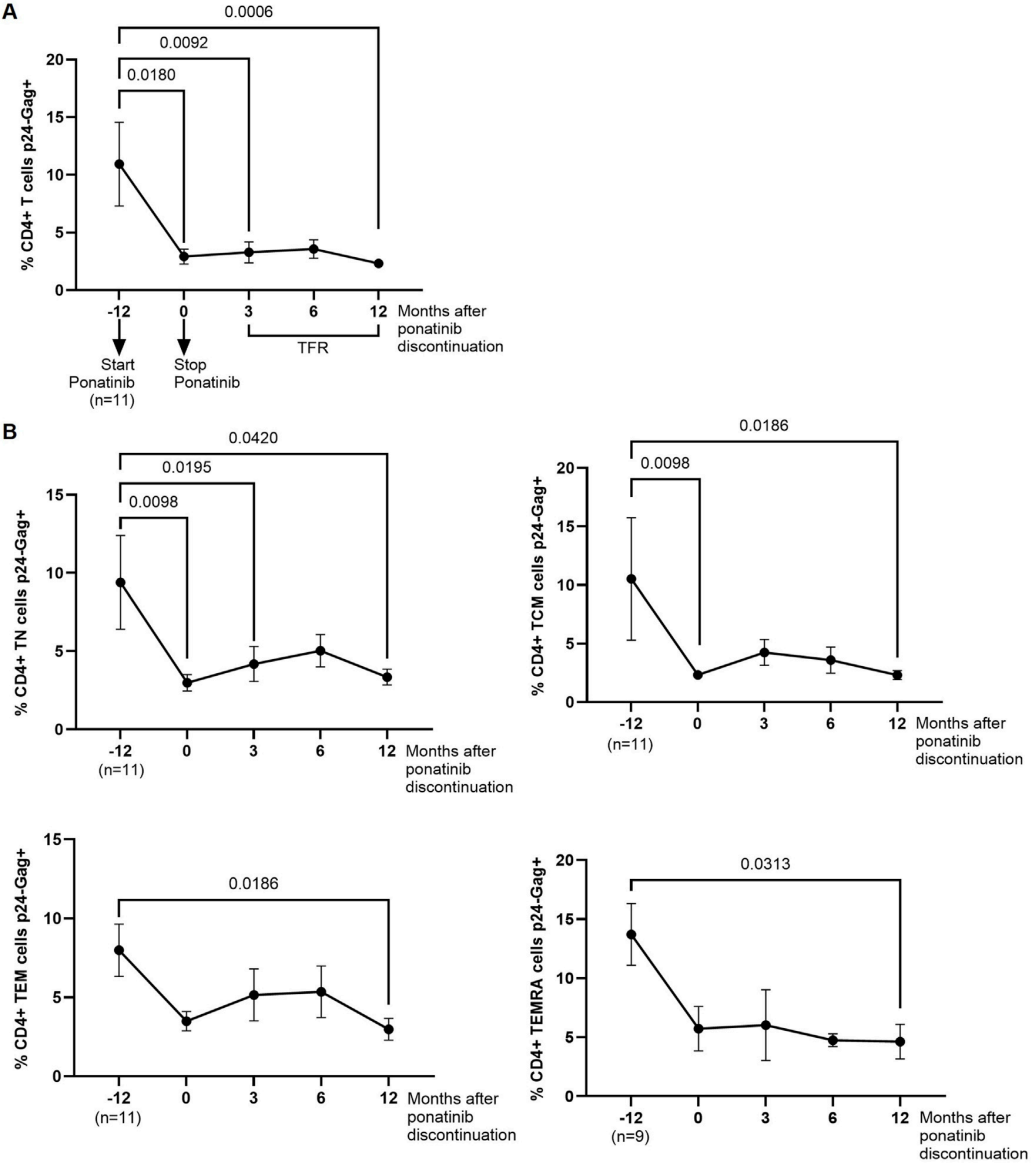
Intracellular levels of p24-gag in CD4^+^ T cells before, during and after treatment with ponatinib. Analysis by flow cytometry of intracellular levels of HIV-1 p24-gag antigen in total CD4^+^ T cells **(A)** and CD4 memory subpopulations (T Naïve (TN), T Central Memory (TCM), T Effector Memory (TEM), and Terminally Differentiated Effector Memory (TEMRA)) **(B)** 72 h after infection of PBMCs with HIV-1 strain NL4-3_wt. Each dot corresponds to the mean of all samples and vertical lines represent the standard error of the mean (SEM). Statistical analysis was performed with Wilcoxon matched-pairs signed rank test and paired t-test. TFR, Treatment-free remission.

PBMCs from different time points were infected *in vitro* with NL4-3_wt strain and the intracellular levels of p24-gag was measured in CD4^+^ T cells 72 h after the infection. The percentage of p24-gag+ CD4^+^ T cells was 3.7-fold (*p* = 0.0180) lower after 12 months of treatment with ponatinib in comparison with previous treatment with imatinib (Figure 1A). These levels were still significantly lower 3 and 12 months after ponatinib discontinuation (3.7-fold, *p* = 0.0092 and 4.7-fold, *p* = 0.0006, respectively). When CD4 memory subpopulations were analyzed, the percentage of CD4^+^ TN and TCM cell subsets that were p24-gag+ during treatment with ponatinib was significantly lower (−3.2-fold, *p* = 0.0098 and −4.5-fold, *p* = 0.0098, respectively), in comparison with previous treatment with imatinib (Figure 1B). 12 months after ponatinib discontinuation, the percentage of p24-gag+ CD4^+^ T cells was significantly lower in all subsets: TN (−2.8-fold; *p* = 0.0420), TCM (−4.5-fold; *p* = 0.0186), TEM (−2.7-fold; *p* = 0.0186), and TEMRA (−3.0-fold; *p* = 0.0313).

### 3.3 Phosphorylation of SAMHD1 at T592 was reduced in CD4^+^ T cells after treatment with ponatinib

**FIGURE 2:**
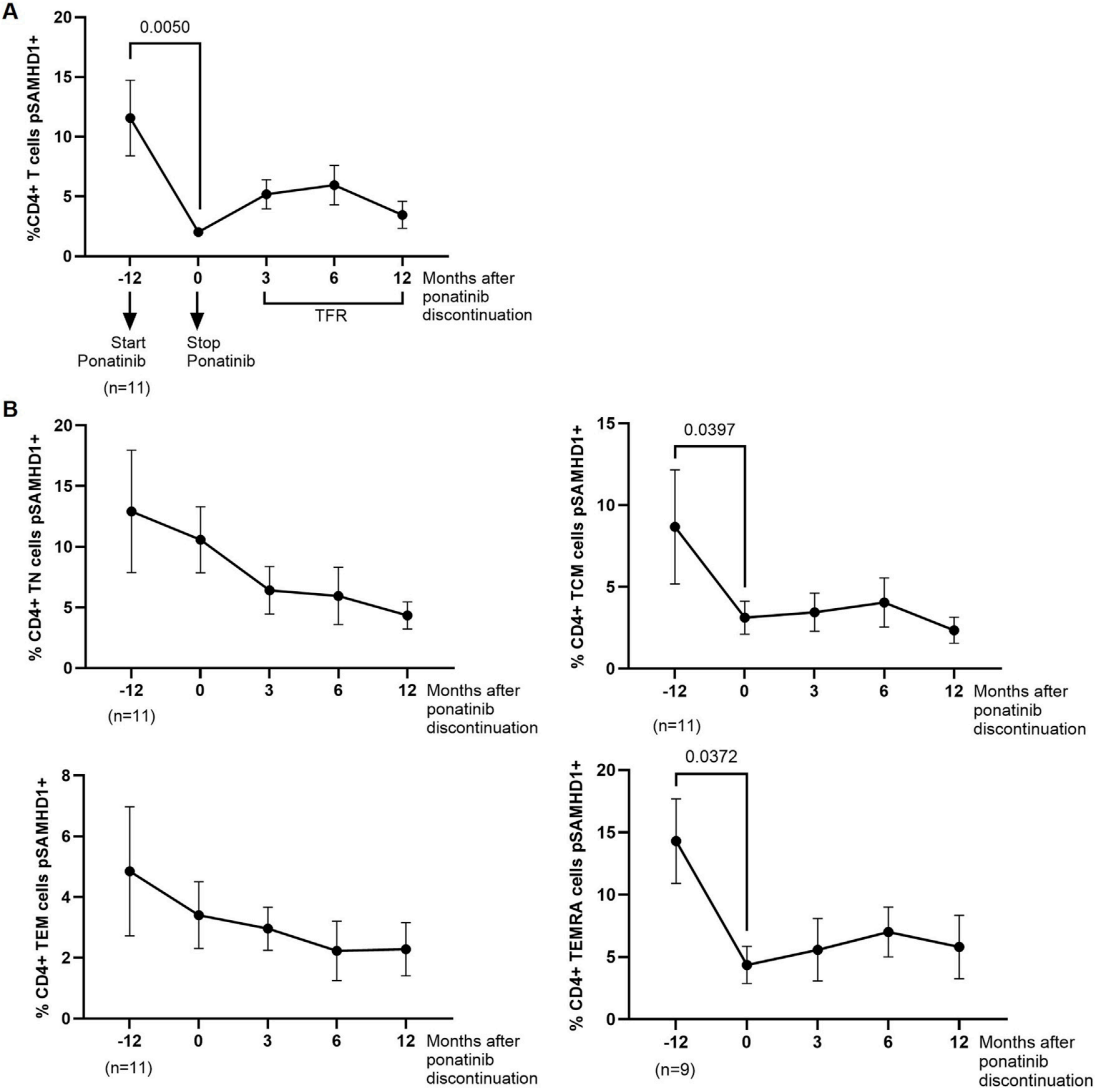
SAMHD1 phosphorylation in CD4^+^ T cells before, during and after treatment with ponatinib. Analysis by flow cytometry of intracellular pSAMHD1 in total CD4^+^ T cells **(A)** and CD4^+^ memory subpopulations (T näive (TN), T central memory (TCM), T effector memory (TEM), and terminally differentiated effector memory (TEMRA)) **(B)** 72 h after infection of PBMCs with HIV-1 strain NL4-3_wt. Each dot corresponds to the mean of all samples and vertical lines represent the SEM. Statistical analysis was performed with Wilcoxon matched-pairs signed rank test and paired t-test. TFR, Treatment-free remission.

pSAMHD1 was analyzed in PBMCs from different time points. During treatment with ponatinib, the percentage of total CD4^+^ T cells expressing pSAMHD1 was 5.7-fold lower (*p* = 0.0050) than during treatment with imatinib (Figure 2A). In CD4 memory subpopulations, significantly lower percentage of TCM and TEMRA subsets pSAMHD1+ were observed during treatment with ponatinib in comparison with previous treatment with imatinib (2.8-fold; *p* = 0.0397 and 3.3-fold; *p* = 0.0372, respectively) (Figure 2B).

### 3.4 Higher cytotoxicity against HIV-1-infected cells after treatment with ponatinib

**FIGURE 3:**
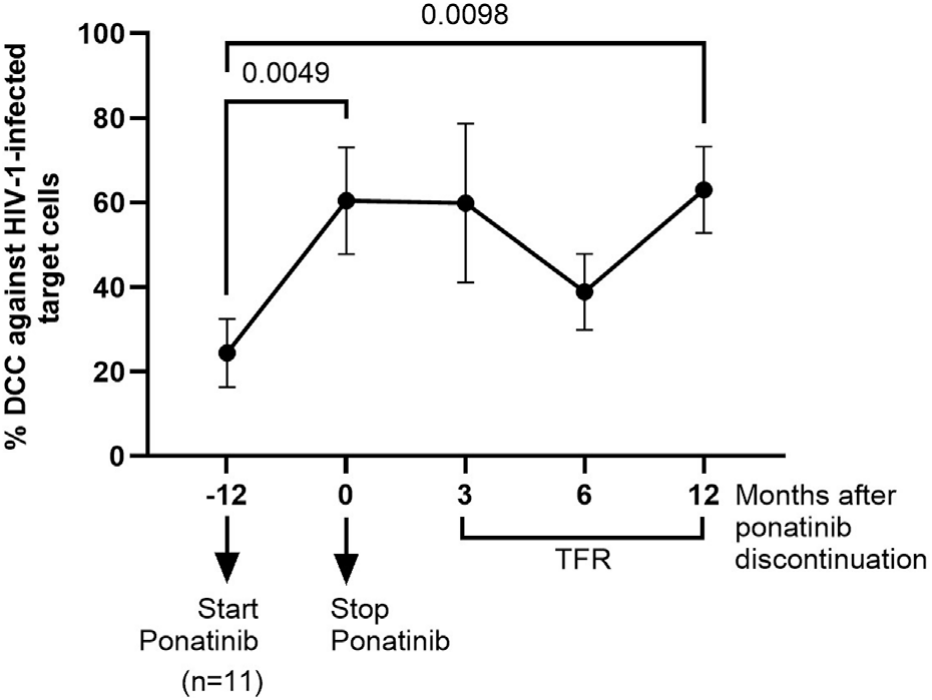
Measurement of DCC of PBMCs against HIV-1-infected target cells before, during and after treatment with ponatinib. DCC was analyzed by measuring caspase-3 activity in a monolayer of HIV-infected TZM-bl cells after co-cultured with PBMCs. Each dot corresponds to the mean of all samples and vertical lines represent the SEM. Statistical analysis was performed with Wilcoxon matched-pairs signed rank test. TFR, Treatment-free remission.

The capacity of PBMCs from different time points to induce DCC in HIV-1-infected TZM-bl cells was measured after co-culture for 1 h. PBMCs obtained after 12 months of treatment with ponatinib showed 2.5-fold (*p* = 0.0049) higher capacity to induce DCC on target cells than PBMCs from the participants when they were still on treatment with imatinib (Figure 3). DCC was still significantly higher in PBMCs obtained 12 months after ponatinib discontinuation (2.6-fold; *p* = 0.0098).

### 3.5 Correlation between p24-gag, pSAMHD1, and DCC after treatment with ponatinib

**FIGURE 4:**
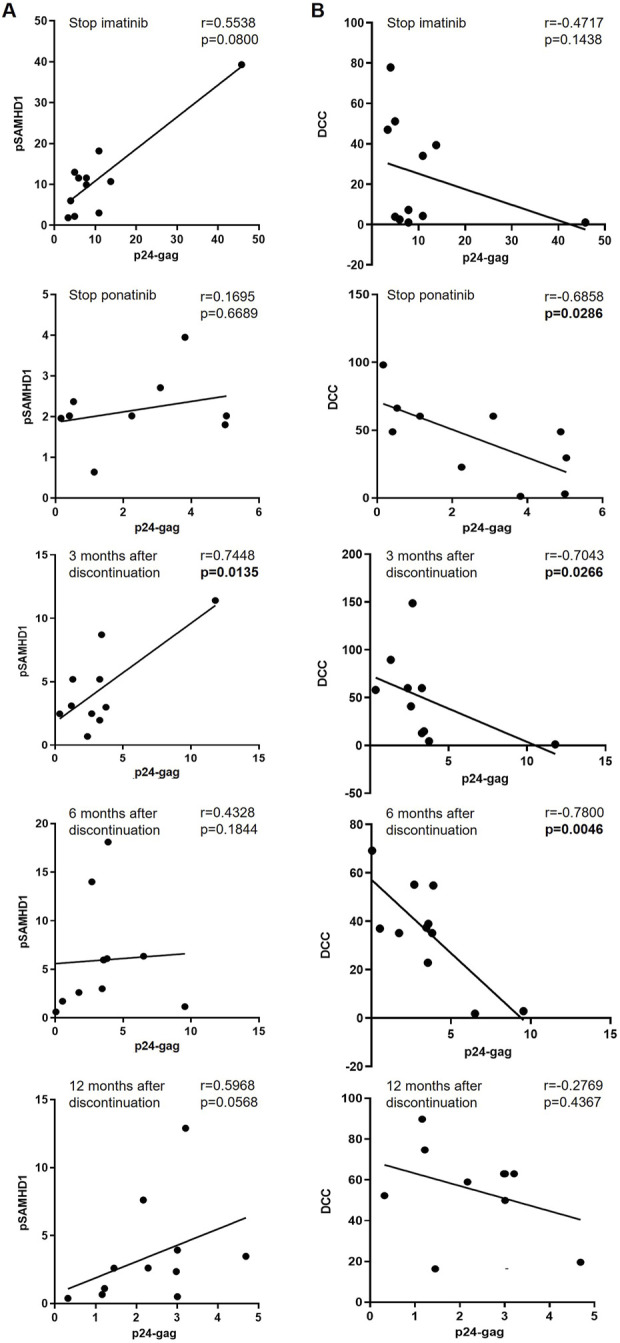
Association between the intracellular levels of p24-gag and pSAMHD1 and DCC in total CD4^+^ T cells before, during and after treatment with ponatinib. Pearson’s or Spearman’s rank correlation coefficients (r) were applied, depending on data normality, to calculate the association between the levels of p24-gag and pSAMHD1 **(A)** or DCC **(B)** of PBMCs from different time points: when treatment with imatinib was stopped (Stop imatinib); when treatment with ponatinib was stopped after 12 months (Stop ponatinib); and 3, 6, and 12 months after ponatinib discontinuation. Each dot corresponds to one sample. Straight line on the graph represents simple linear regression. *p*-values showing statistical significance (*p* < 0.05) are highlighted in bold letters.

Although there was no significant correlation between the intracellular levels of p24-gag and pSAMHD1 in CD4^+^ T cells when the participants were on treatment with imatinib (Figure 4A), we observed a positive correlation between these parameters in CD4^+^ T cells 3 months after discontinuation of ponatinib (r = 0.7448; *p* = 0.0135). In the analysis of the association between the intracellular levels of p24-gag in CD4^+^ T cells and the cytotoxic activity (DCC) of PBMCs against HIV-1-infected target cells, there was a significant negative correlation when cells were obtained during treatment with ponatinib (r = −0.6858; *p* = 0.0286), and 3 and 6 months after ponatinib discontinuation (r = −0.7043, *p* = 0.0266 and r = −0.7800, *p* = 0.0046) (Figure 4B).

### 3.6 Changes in cytotoxic cell populations in response to treatment with ponatinib

**FIGURE 5:**
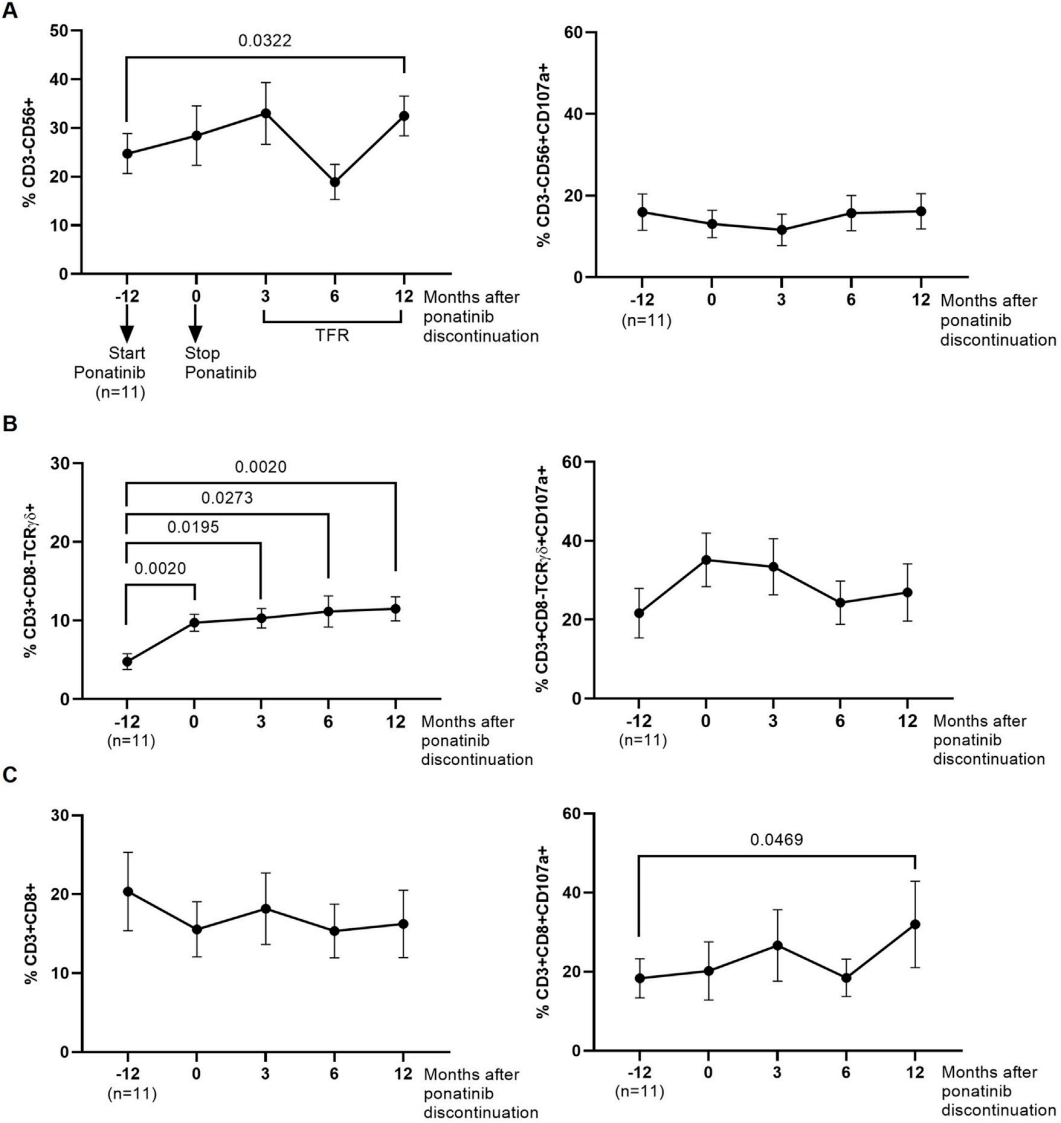
Phenotypic analysis of cytotoxic cells that were co-cultured with the monolayer of HIV-1-infected TZM-bl cells. Levels of NK cells (CD3^−^CD56^+^) **(A)**, CD8^+^ T cells (CD3^+^CD8^+^) **(B)**, and Tγδ cells (CD3^+^CD8-TCRγδ+) **(C)**, as well as their degranulation capacity (CD107a+), were analyzed by flow cytometry. Each dot corresponds to the mean of all samples and vertical lines represent the SEM. Statistical analysis was performed with Wilcoxon matched-pairs signed rank test and paired t-test. TFR, Treatment-free remission.

We analyzed by flow cytometry the presence of cell populations with cytotoxic activity that could be responsible for inducing DCC on HIV-1-infected TZM-bl target cells. The levels of NK cells (CD3^−^CD56^+^) were 1.3-fold (*p* = 0.0322) higher in PBMCs obtained 12 months after ponatinib discontinuation than when PBMCs were obtained while on treatment with imatinib (Figure 5A, left graph). The degranulation capacity of these cells, measured by the levels of CD107a, was not modified during treatment with ponatinib or after discontinuation (Figure 5A, right graph).

The levels of Tγδ cells with phenotype CD3^+^CD8-TCRγδ+ in PBMCs obtained during treatment with ponatinib and 3, 6, and 12 months after discontinuation were higher than in PBMCs obtained during treatment with imatinib (2.0-fold, *p* = 0.0020; 2.1-fold, *p* = 0.0195; 2.3-fold, *p* = 0.0273; and 2.4-fold, *p* = 0.0020, respectively) (Figure 5B, left graph). No significant changes were observed in the levels of CD107a on the surface of these cells (Figure 5B, right graph). The levels of Tγδ cells with phenotype CD3^+^CD8+TCRγδ+ or the levels of CD107a were not modified during or after treatment with ponatinib (Supplementary Figure S3).

No significant changes were observed in the levels of CD8^+^ T cells in PBMCs from different time points (Figure 5C, left graph), but the levels of CD107a+ was higher in CD8^+^ T cells from PBMCs obtained 12 months after ponatinib discontinuation than in PBMCs while still on treatment with imatinib (1.7-fold; *p* = 0.0469) (Figure 5C, right graph).

### 3.7 DCC against autologous HIV-1-infected CD4^+^ T cells

**FIGURE 6:**
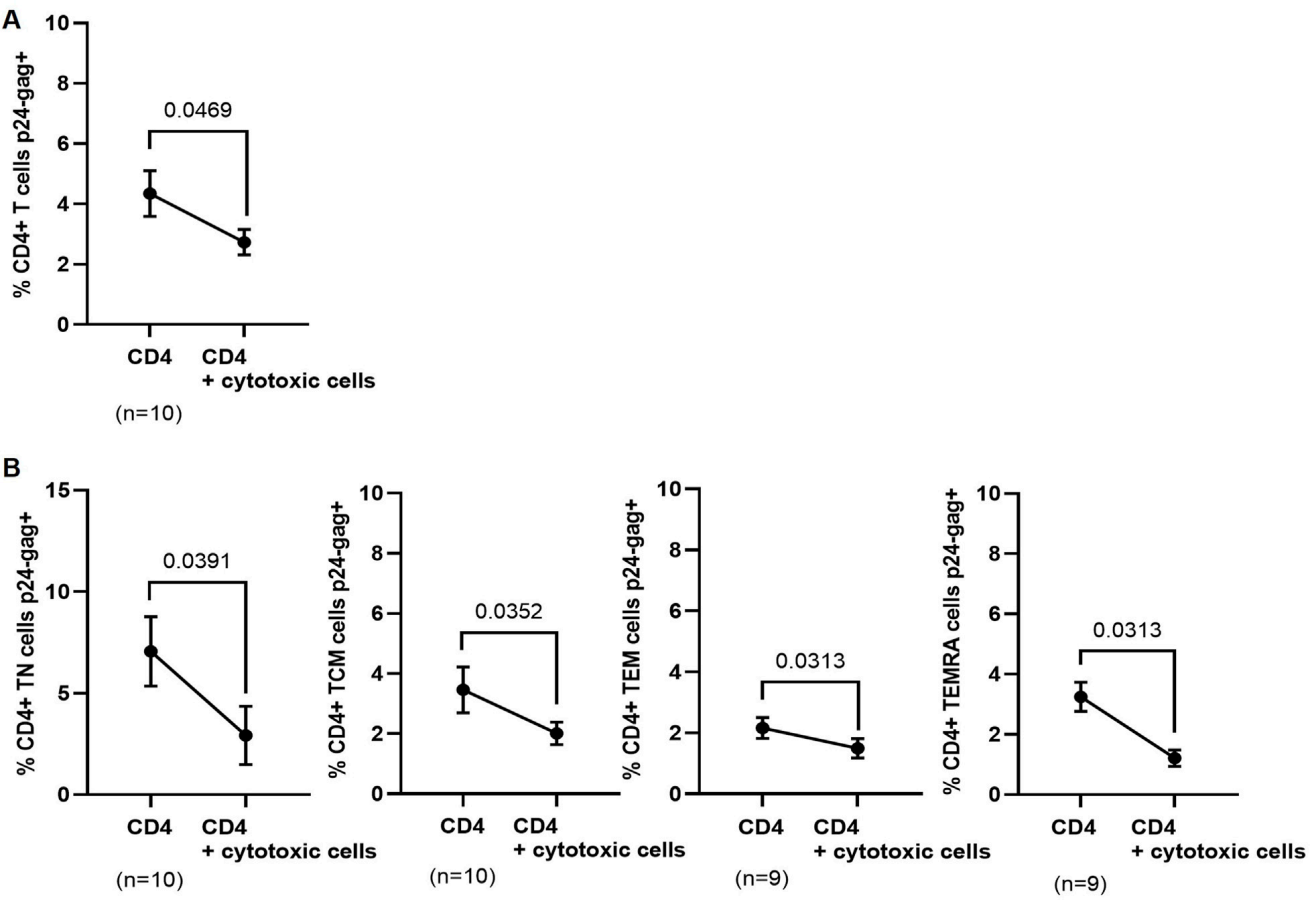
Levels of p24-gag+ CD4^+^ T cells after co-culture with autologous cytotoxic cell populations obtained 12 months after ponatinib discontinuation. Levels of total p24-gag+ CD4^+^ T cells **(A)** or p24-gag + CD4 memory subpopulations **(B)** that were infected with NL4-3_wt strain in the presence or absence of autologous cytotoxic populations for 72 h. Each dot corresponds to the mean of all samples and vertical lines represent the SEM. Statistical analysis was performed with paired t-test.

We analyzed the direct cytotoxic capacity (DCC) of PBMCs obtained 12 months after ponatinib discontinuation to reduce the percentage of autologous CD4^+^ T cells expressing p24-gag during co-culture. CD4^+^ T cells were isolated from PBMCs and then infected with NL4-3_wt strain in the presence or absence of autologous cytotoxic cells. The levels of total p24-gag+ CD4^+^ T cells were 1.6-fold lower (*p* = 0.0469) when they were co-cultured with autologous cytotoxic cells for 72 h, in comparison with the same CD4^+^ T cells infected alone (Figure 6A). When CD4 memory subsets were analyzed, the levels of all p24-gag + subpopulations were lower when they were co-cultured with autologous cytotoxic cells than when they were cultured alone: TN (2.4-fold; *p* = 0.0391); TCM (1.7-fold; *p* = 0.0352); TEM (1.4-fold; *p* = 0.0313), and TEMRA (2.7-fold; *p* = 0.0313) cells (Figure 6B).

## 4 Discussion

Different strategies have been designed and implemented to produce a substantial effect on HIV-1 long-lived, latent reservoir. Although promising, approaches such as LRAs (Martrus and Altfeld, 2016) or latency-promoting agents (LPAs) (Vargas et al., 2019) have not led to significant changes in the viral reservoir *in vivo* so far (Thomas et al., 2020; Kuang and Brockman, 2018; Ait-Ammar et al., 2019), while other successful strategies such as allogenic transplantation of bone marrow stem cells (HSCT) from homozygous CCR5Δ32 donors (Gupta et al., 2019; Hsu et al., 2023; Hütter et al., 2009) cannot be used for all PWH. HIV-1 cure strategies include both remission and eradication and the development of a scenario of remission in which the virus located in hidden sanctuaries may be controlled by the immune system in the absence of ART seems more realistic than the complete viral eradication by a dramatic intervention such as HSCT (Deeks et al., 2021). Therefore, strategies aimed at stimulating the antiviral immune response in PWH are essential toward achieving remission, maybe combined with LPAs.

The use of immunomodulators to interfere with HIV-1 reservoir formation and replenishment has been under investigation for some time, such as the use of IL-7 for its potential to enhance T-cell survival and proliferation with the objective to impact HIV reservoir size, but that enhanced HIV-1 persistence during ART instead (Vandergeeten et al., 2013); immune checkpoint inhibitors, such as anti-PD-1 and anti-CTLA-4 antibodies, to induce the reactivation of the reservoirs and to stimulate the immune-mediated clearance of infected cells (Benito et al., 2023); toll-like receptor (TLR) agonists to stimulate the innate immune response and reduce the viral reservoir (Board et al., 2022); as well as therapeutic vaccines, broadly neutralizing antibodies (bNAbs), and other immune-based strategies, to target and reduce HIV reservoirs (Haynes et al., 2023).

TKIs are also potential agents to interfere with HIV-1 persistence that may favor the prospect for viral eradication (Rodríguez-mora et al., 2019). In this regard, dasatinib and ponatinib are good candidates to be used in combination with ART with the aim to interfere with the replenishment and maintenance of HIV-1 reservoir (Bermejo et al., 2018; Innis et al., 2021; Salgado et al., 2019). Based on previous results obtained by our group, three Phase II, randomized clinical trials are currently ongoing in United States and Spain with PWH who will receive dasatinib to test safety and efficacy to interfere with the reservoir formation and maintenance as primary endpoints (NCT05527418, NCT05780073, and NIH ACTG trial A5413). Ponatinib is a pan-BCR::ABL1 inhibitor approved by the FDA in 2012 for treating CML in individuals who developed T315I mutation that is resistant to previous TKIs (Shah, 2011) and presents a similar antiviral profile than dasatinib (Bermejo et al., 2018). The marketing approval was suspended in 2013 due to safety concerns primarily related to ischemic events. However, the suspension was lifted the same year after the implementation of new safety measures such as the adjustment of the recommended dose to 30–45 mg daily, being 15 mg daily the lower effective dose (Jabbour and Kantarjian, 2018). In this substudy of NCT04043676, all participants showed good tolerance to 15 mg of ponatinib daily for 1 year and the safety profile was adequate, although several adverse events were reported that were similar to those observed in the general cohort such as constipation, asthenia, myalgias, arthralgias, and skin rash (Pérez-Lamas et al., 2023). Treatment with ponatinib for 1 year did not produce lymphopenia or change CD4^+^ T−cell levels in the participants of the study. These cells were susceptible to HIV-1 infection while they were on treatment with imatinib, in correlation with an increase in SAMHD1 phosphorylation in response to T-cell activation, as previously described (Bermejo et al., 2018). However, CD4^+^ T cells showed resistance to HIV-1 infection after 1-year treatment with ponatinib. All CD4 memory subpopulations expressed low levels of intracellular p24-gag in response to HIV-1 infection during treatment with ponatinib. Although HIV-1 reservoir can be found in higher proportion in TCM and TEM cells than TN and TEMRA (Chomont et al., 2009; Buzon et al., 2014; Jaafoura et al., 2014), TEMRA cells contribute more efficiently to the reservoir replenishment and proviral rebound since they contain a higher proportion of unstable viral forms (Trémeaux et al., 2019). Therefore, TEM and TEMRA are essential targets to tackle the viral reservoir. Nevertheless, the fact that resistance to HIV-1 infection was observed in all CD4 cell subpopulations suggested that ponatinib could interfere with the establishment, replenishment, and proliferation of HIV-1 reservoir if used as adjuvant of ART in PWH. Ponatinib interferes with CD4^+^ T−cell activation and proliferation driven by homeostatic cytokines and TCR-mediated activation (Innis et al., 2021) and it also impedes HIV-1 proviral integration in CD4^+^ T cells (Bermejo et al., 2018). Through the interference with CD4 proliferation, ponatinib could inhibit the reservoir replenishment and reduce the reservoir size in PWH, as was described for dasatinib (Vigón et al., 2021). In addition, ponatinib may also impede the production of proteins from defective integrated proviruses that contribute to antigen-driven activation of the immune system, which leads to chronic inflammation (Imamichia et al., 2016). Therefore, ponatinib may display an anti-inflammatory potential similar to that of dasatinib, whose senotherapeutic properties have already been evaluated in several clinical trials (Justice et al., 2019; Saccon et al., 2021; Zeng et al., 2019).

We did not find a significant correlation between the reduced intracellular levels of p24-gag and the preservation of SAMHD1 antiviral activity, which appointed at the existence of an additional antiviral mechanism triggered by treatment with ponatinib that may be sustained even during TFR. In fact, CD4^+^ T cells isolated from individuals with CML who discontinued treatment with dasatinib regained the susceptibility to be infected with HIV-1 after 1 year since discontinuation and SAMHD1 was phosphorylated upon activation in these cells (Vigón et al., 2020). Recently, it has been described that ponatinib may interfere with AKT/mTOR signaling pathway, blocking HIV-1 proviral reactivation from latency (Huang et al., 2023). However, this effect could be similar to the interference with SAMHD1 phosphorylation, and it will likely be effective only during treatment and be recovered after discontinuation.

The antiviral effect exerted by ponatinib seemed to be active on CD4^+^ T cells until the end of the follow-up 12 months since ponatinib discontinuation. It has been described that treatment with TKIs may induce the development of long-term cytotoxic cell populations with degranulation capacity (Kreutzman et al., 2010; Hayashi et al., 2012; Mustjoki et al., 2013; Vigón et al., 2020; Lachota et al., 2018). Moreover, we previously reported that individuals with CML on TFR after discontinuation of dasatinib showed potent cytotoxicity against autologous HIV-1-infected CD4^+^ T cells (Vigón et al., 2020). Our results indicated that increased cytotoxicity of PBMCs after treatment with ponatinib relied on higher levels of cytotoxic cells such as NK and Tγδ cells that were able to reduce the levels of autologous p24-gag+ CD4^+^ T cells. In fact, ponatinib regulates NK cell function through IL-15 signaling pathway (Harrington et al., 2020), which also regulates Tγδ cell activity (Van Acker et al., 2016). The essential role of NK cells in the development of cytotoxic activity against HIV-1-infected CD4^+^ T cells has been previously described for dasatinib (Vigón et al., 2020), but this is the first report about the effect of ponatinib on Tγδ cell count. Interestingly, despite the cytostatic effect of ponatinib based on the inhibition of IL-2-mediated T-cell proliferation, we did not observe a significant reduction in the total percentage of CD8^+^ T cells during and after treatment with ponatinib, as occurred with CD4^+^ T cells. Moreover, CD8^+^ T cells displayed higher degranulation capacity 12 months after treatment with ponatinib.

In our cohort, most individuals were seropositive to CMV, but they did not show signs of immunosuppression or CMV reactivation. The enhanced cytotoxic activity in people with CML could be responsible for the absence of reactivation of endogenous herpesviruses such as CMV despite the cytostatic effect of TKIs. On the contrary, the presence of CMV may co-stimulate TKI-induced immune response to contribute to the development of LGLs with both antileukemic and antiviral activity (Kreutzman et al., 2011), which may support the effect against HIV-1 showed by PBMCs isolated from these individuals. Potentially improved cytotoxicity against cancerous cells has been previously described for dasatinib (Mustjoki et al., 2013; Chang et al., 2018; Hassold et al., 2012) and imatinib (Hayashi et al., 2012), but not for ponatinib. In our cohort, the cytotoxic activity stimulated by ponatinib was active to protect CD4^+^ T cells from HIV-1 infection even 1 year since treatment discontinuation. Interestingly, this protection was significantly higher in TEMRA subpopulation, which is a major contributor to the reservoir replenishment, as described above (Trémeaux et al., 2019).

In addition to TKIs, other immunodulators have been described with potential to interfere with HIV-1 infection and reservoir formation. Specifically, JAK1/2 inhibitors have shown antiviral and anti-inflammatory effects (Yu et al., 2024) and some of them such as baricitinib or ruxolitinib have been proposed to target the HIV-1 reservoir (de Armas et al., 2021). Ruxolitinib is an FDA-approved agent for treating myelofibrosis and rheumatoid arthritis with capacity to selectively inhibit HIV-1 replication in lymphocytes and macrophages (Gavegnano et al., 2014; Gavegnano et al., 2017; Reece et al., 2022). AIDS Clinical Trial (ACTG) A5336 sponsored open-label randomized Phase 2a multi-site trial has shown promising results about the efficacy of ruxolitinib to induce a progressive decay of the HIV-1 reservoir and to reset the immune balance in PWH on ART (Marconi et al., 2022; Reece et al., 2024). Moreover, treatment with this JAK inhibitor has been related to a possible case of an HIV cure in the “Geneva patient” who received a stem cell transplant from a CCR5 wild-type donor to treat biphenotypic sarcoma (Sáez-Cirión et al., 2023). Former cases of HIV cure following stem cell transplant involved donors who were homozygous for the CCR5Δ32 mutation (Hsu et al., 2023), while PWH such as the “Boston patients” who received wild type stem cell transplants presented viral rebound few months after ART discontinuation (Henrich et al., 2014). In the “Geneva patient”, several factors may have contributed to the HIV cure or at least extended remission, such as the use of ruxolitinib to facilitate the transplant that may have also reduced the possibility of the virus infecting new target cells from the donor. Baricitinib is also currently under investigation to test its safety and efficacy to decrease the HIV reservoir in the central nervous system in PWH with stable virologic suppression on ART (NCT05452564).

In conclusion, new strategies aimed at promoting viral remission in the absence of ART are necessary to advance towards an HIV-1 cure. The transient use of immunomodulatory drugs able to reprogram the immune response to make it more efficient against HIV-1-infected CD4^+^ T cells could be effective in combination with ART to promote a persistent antiviral state that might contribute to control viral rebound after ART discontinuation. Intensification treatment with a low and safe dose of ponatinib was effective as LPA to protect CD4^+^ T cells during treatment due to its cytostatic activity, mostly by preserving SAMHD1 antiviral activity, but also after discontinuation by inducing long-term cytotoxic populations which correlated with a decrease in the intracellular levels of viral proteins such as p24-gag from HIV-1-infected CD4^+^ T cells. Although these data give hope about the possibility of repositioning TKIs such as ponatinib to enhance the antiviral activity of PBMCs in PWH on ART as a new strategy towards HIV functional cure, we cannot rule out that this enhanced cytotoxicity induced by ponatinib may also be triggered and sustained by the presence of cancerous cells in people with CML that may be stimulating the immune response, similarly to CMV. Other potential limitation of this study is that we did not explore the underlying molecular mechanism that made possible the reprogramming of the cytotoxic cells. In addition, we did not quantify the proviral integration due to lack of sample, but we used the measurement of intracellular p24-gag by flow cytometry instead, which has been previously described as a valid quantification assay to monitor the productive HIV-1 reservoir during the standardization of cure strategies (Puertas et al., 2021). Consequently, more studies are necessary to unravel the mechanisms that mediate the immunomodulatory effect of TKIs. Nevertheless, this pilot study proved that short-term treatment with immunomodulatory agents may represent an alternative method toward progressively reprogramming the immune system against the viral reservoir, paving the way to perform clinical trials with PWH.

## Data Availability

The original contributions presented in the study are included in the article/Supplementary Material, further inquiries can be directed to the corresponding author.
