# Supplementary material for: Sustained antiviral response against in vitro HIV-1 infection in peripheral blood mononuclear cells from people with chronic myeloid leukemia treated with ponatinib
Source: Front Pharmacol. 2024 Sep 23;15:1426974. doi: 10.3389/fphar.2024.1426974 (PMC11460598; doi:10.3389/fphar.2024.1426974)
Supplement: Supplementary file 1 [file Presentation1.PPTX]

## Slide 1
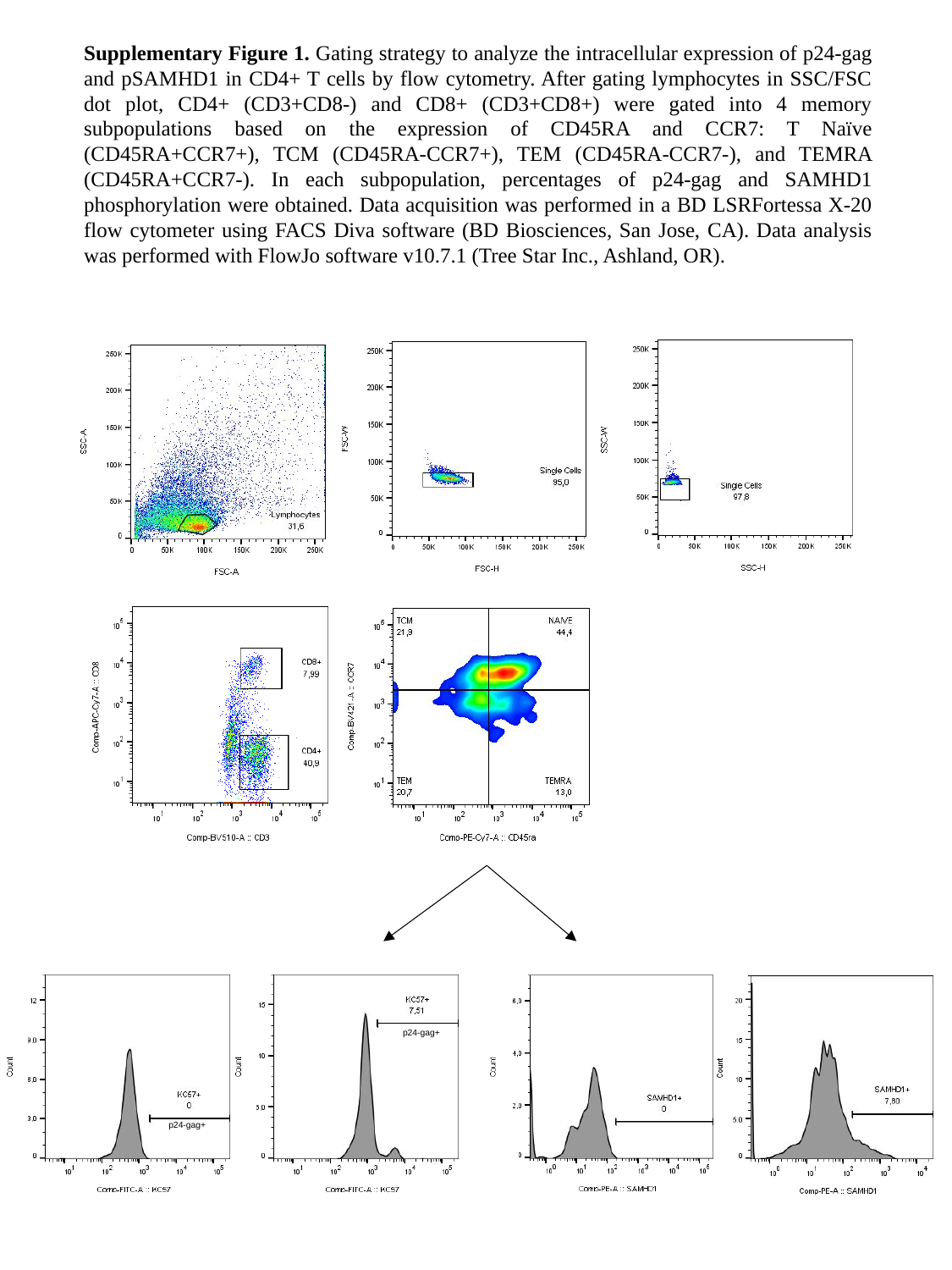

Supplementary Figure 1. Gating strategy to analyze the intracellular expression of p24-gag and pSAMHD1 in CD4+ T cells by flow cytometry. After gating lymphocytes in SSC/FSC dot plot, CD4+ (CD3+CD8-) and CD8+ (CD3+CD8+) were gated into 4 memory subpopulations based on the expression of CD45RA and CCR7: T Naïve (CD45RA+CCR7+), TCM (CD45RA-CCR7+), TEM (CD45RA-CCR7-), and TEMRA (CD45RA+CCR7-). In each subpopulation, percentages of p24-gag and SAMHD1 phosphorylation were obtained. Data acquisition was performed in a BD LSRFortessa X-20 flow cytometer using FACS Diva software (BD Biosciences, San Jose, CA). Data analysis was performed with FlowJo software v10.7.1 (Tree Star Inc., Ashland, OR).
p24-gag+
p24-gag+
