# Supplementary material for: Sustained antiviral response against in vitro HIV-1 infection in peripheral blood mononuclear cells from people with chronic myeloid leukemia treated with ponatinib
Source: Front Pharmacol. 2024 Sep 23;15:1426974. doi: 10.3389/fphar.2024.1426974 (PMC11460598; doi:10.3389/fphar.2024.1426974)
Supplement: Supplementary file 2 [file Presentation3.PPTX]

## Slide 1
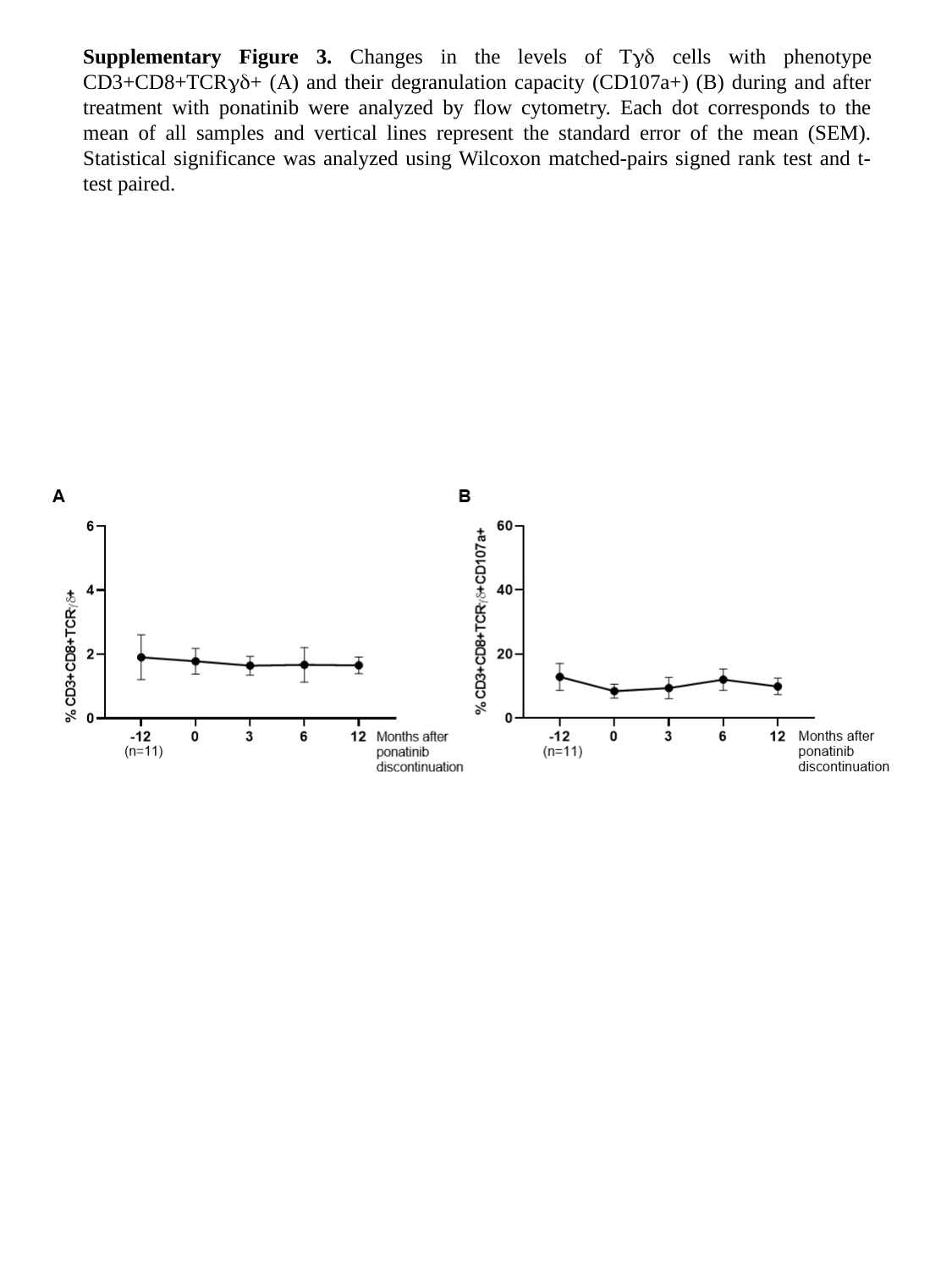

Supplementary Figure 3. Changes in the levels of Tgd cells with phenotype CD3+CD8+TCRgd+ (A) and their degranulation capacity (CD107a+) (B) during and after treatment with ponatinib were analyzed by flow cytometry. Each dot corresponds to the mean of all samples and vertical lines represent the standard error of the mean (SEM). Statistical significance was analyzed using Wilcoxon matched-pairs signed rank test and t-test paired.
