# Supplementary material for: Sustained antiviral response against in vitro HIV-1 infection in peripheral blood mononuclear cells from people with chronic myeloid leukemia treated with ponatinib
Source: Front Pharmacol. 2024 Sep 23;15:1426974. doi: 10.3389/fphar.2024.1426974 (PMC11460598; doi:10.3389/fphar.2024.1426974)
Supplement: Supplementary file 3 [file Presentation2.PPTX]

## Slide 1
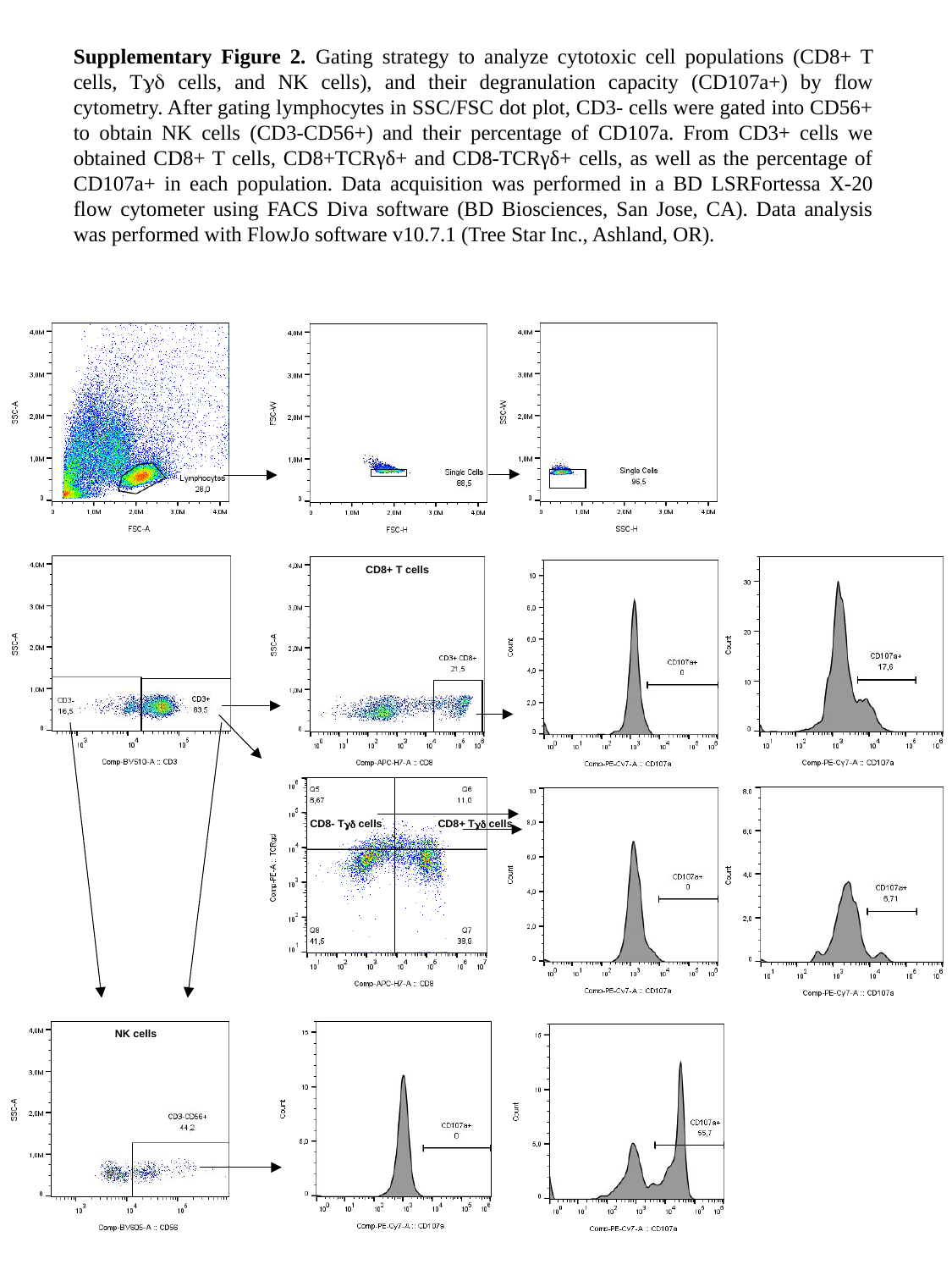

Supplementary Figure 2. Gating strategy to analyze cytotoxic cell populations (CD8+ T cells, Tgd cells, and NK cells), and their degranulation capacity (CD107a+) by flow cytometry. After gating lymphocytes in SSC/FSC dot plot, CD3- cells were gated into CD56+ to obtain NK cells (CD3-CD56+) and their percentage of CD107a. From CD3+ cells we obtained CD8+ T cells, CD8+TCRγδ+ and CD8-TCRγδ+ cells, as well as the percentage of CD107a+ in each population. Data acquisition was performed in a BD LSRFortessa X-20 flow cytometer using FACS Diva software (BD Biosciences, San Jose, CA). Data analysis was performed with FlowJo software v10.7.1 (Tree Star Inc., Ashland, OR).
CD8+ T cells
CD8- Tgd cells
CD8+ Tgd cells
NK cells
